# Inhibition of the IGF signaling pathway reverses cisplatin resistance in ovarian cancer cells

**DOI:** 10.1186/s12885-017-3840-1

**Published:** 2017-12-14

**Authors:** Juan Du, Hui-rong Shi, Fang Ren, Jing-lu Wang, Qing-hua Wu, Xia Li, Rui-tao Zhang

**Affiliations:** Department of Obstetrics and Gynecology, The First Affiliated Hospital of Zhengzhou University, Zhengzhou, 450052 Henan Province People’s Republic of China

**Keywords:** Cisplatin, DNA repair, Drug resistance, Hematoxylin and eosin staining, Immunohistochemistry, Insulin-like growth factor signaling pathway, Metformin, *MRP2*, Ovarian tumor, Tumor-inhibition rate

## Abstract

**Background:**

This study was aimed at investigating whether metformin can reverse the resistance of ovarian cancer cells to cisplatin and exploring the underlying mechanism.

**Methods:**

Ovarian cancer cell proliferation in vitro was evaluated using a CCK-8 assay. The resistance index of platinum-resistant ovarian cancer cells was determined and cell cycle and apoptosis rate determined by annexin V/propidium iodide double-staining in CP70 cells. Western blotting was used to determine IGF1, IGF1R, AKT, p-IGF1, p-IGF1R, p-AKT, and MRP2 levels in cells treated with different concentrations of metformin and LY29400, an inhibitor of the insulin-like growth factor pathway. Changes in gene expression levels of *MRP2*, *IGF1*, *IGF1R*, and *AKT* were determined by fluorescence real-time quantitative PCR assay of CP70 cells treated with metformin. Tumors of human ovarian cancer cell lines CP70 and A2780 were established by subcutaneous transplantation of cells in nude mice and the effect of metformin on MRP2 expression and tumor inhibition assessed.

**Results:**

The IC_50_ value of cisplatin in CP70 cells decreased significantly as metformin concentration increased (*P* < 0.05). The cell cycle distribution in CP70 cells changed with metformin treatment; the percentage of cells in the G0/G1 phase, as well as the natural apoptosis rate was significantly increased with metformin treatment (*P* < 0.05). IGF1, IGF1R, AKT p-IGF1, p-IGF1R, and p-Akt protein expression was enhanced dose-dependently with metformin, and was also significantly changed by treatment of CP70 cells with 0 mM metformin +10 mM LY294002. Moreover, changes in the expression of *MRP2*, *IGF1*, *IGF1R,* and *AKT* was metformin-concentration dependent, and was significantly different from that in the untreated control group (*P* < 0.05). In nude mice, the tumor volumes of the cisplatin-treated groups were significantly less than in the control group, and was further suppressed by co-treatment with cisplatin and metformin (*P* < 0.05), indicating that these 2 drugs had a synergistic effect on tumor inhibition.

**Conclusion:**

Metformin can improve the sensitivity of ovarian cancer CP70 cells to cisplatin in a concentration-dependent manner by activating the AKT signaling pathway, inhibiting the IGF1R signaling pathway, and reducing MRP2 expression.

**Electronic supplementary material:**

The online version of this article (10.1186/s12885-017-3840-1) contains supplementary material, which is available to authorized users.

## Background

Metformin is an agent used for increasing insulin-sensitivity in the treatment of type II diabetes. In recent years, it has been shown that metformin also plays a role in the prevention and treatment of cardiovascular disease, polycystic ovary syndrome, and tumors [1, 2]. In patients with diabetes, it has been suggested that metformin use prevents cancer development, and there is a lot of interest in whether the metabolic regulation of metformin may also have a role to play in cancer prevention in patients without diabetes [3].

Among gynecologic malignancies, ovarian cancer has the highest mortality rate; in women, mortality due to this cancer is the fifth highest. Most of these patients are identified too late, when surgical treatment is unlikely to be curative. Platinum-based combination chemotherapy is one of the important means of comprehensive treatment for ovarian epithelial cancer [4]. Cisplatin (DDP) leads to formation of DNA adducts and causes DNA damage in tumor cells, inhibiting tumor cell DNA replication and cell division, and eventually killing tumor cells. However, the efficacy of cisplatin is often reduced because cancer cells acquire drug resistance; this is the main reason for treatment failure in patients.

Ovarian cell lines have been useful in exploring the mechanisms of resistance to anti-cancer drugs, such as the ovarian tumor cell line A2780, which although itself sensitive to cisplatin, has been used to isolate cells that are cancer drug resistant. Studies on CP70 cells, a cisplatin resistant cell line derived from A2780, show that they have microsatellite instability, a phenotype designated RER^+^ and they are deficient in mismatch repair [5, 6]. While the mechanism underlying cisplatin resistance in tumors is not fully understood, it is known to involve the nucleotide excision repair (NER) pathway [7]. Excision repair cross-complementation 1 (ERCC1) is one of the most important repair proteins in the NER pathway, which can reduce the sensitivity of tumor cells to platinum-based chemotherapy, suggesting that it can repair platinum-induced DNA damage [8]. Silencing or interfering *ERCC1* expression in ovarian cancer cells can increase cisplatin sensitivity in cells [9]. Additionally, cancer cell lines that are insensitive to cisplatin overexpress multidrug resistance associated protein 2 (MRP2) [10]. When MRP2 levels are reduced by short hairpin RNA in CP70 cells drug sensitivity is restored [11]. MRP2 is one of the ATP-binding cassette super-family of transporters, these proteins efflux cytotoxic agents, including anticancer drugs such as cisplatin [12]. When MRP2 expression is low therefore, cisplatin levels can be seen to accumulate in cells [11]. Metformin has been shown to enhance cisplatin sensitivity in ovarian cancer cells [4]. In this study, we investigated the mechanism by which metformin can reverse the resistance of ovarian cancer cells to cisplatin with particular attention on MRP2. We show that metformin reduces the expression of *MRP2* in ovarian cancer cells, providing novel insights into ovarian cancer chemotherapy.

## Methods

RPMI 1640 cell culture medium and fetal bovine serum (FBS) were purchased from Gibco (Carlsbad, CA, USA). Metformin and cisplatin were purchased from Sigma−Aldrich (St Louis, MO, USA). An inhibitor of the insulin-like growth factor pathway, LY294002, was obtained from Calbiochem (Billerica, MA, USA). Cell count kit-8 (CCK-8) was purchased from Japan Dojindo Laboratories, (Kumamoto, Japan). RIPA cell lysis buffer was purchased from Beijing Solarbio Science & Technology Co. Ltd., (Beijing China). Real-time fluorescence quantitative PCR reagent was bought from Toyobo (Osaka, Japan). Rabbit anti-human MRP2, anti-IGF1, anti-phospho-IGF1, anti-IGF1R, anti-phospho-IGF1R, anti-AKT, and anti-phospho-AKT polyclonal antibodies were purchased from Cell Signaling Technology (Danvers, MA, USA). Rabbit anti-human GAPDH polyclonal antibody was purchased from China Hangzhou Goodhere Biotechnology Co. Ltd., (Hangzhou, China). Horseradish peroxidase-labeled goat anti-rabbit IgG antibody was purchased from EarthOx Life Sciences (Millbrae, CA, USA). Primers used for PCR were made by Sangon Biological Engineering Technology and Service Co. Ltd. (Shanghai, China).

### Cell culture

A2780 cells and a cisplatin-resistant human ovarian cancer cell line CP70 were provide by the reproductive center of the Fourth Military Medical University. Cells were cultured in the RPMI 1640 containing 10% FBS, at 37 °C, in 5% CO_2_, and were conventionally passaged. Cells in logarithmic growth phase were used for experiments.

### Drug preparation

Five grams of metformin (30 mmol) was dissolved in 30 ml of sterile phosphate-buffered saline, and this stock solution (1 mol/L) was stored at −20 °C. Working solutions of the required concentration were prepared in cell culture medium immediately before use in the experiments. It should be noted that the concentrations of metformin were used at a level that exceeds its normal therapeutic plasma concentration (Cmax20μmol/L) [13].

### Cell proliferation and cytotoxicity

A2780 and CP70 cells (7 × 10^3^ cells in 100 μL serum-free culture medium) were plated in 96-well plates and cultured for 24 h. Then, the medium was replaced with RPMI 1640 containing FBS and 1, 2, 4, 8, 16, 32, or 64 μg/mL cisplatin, and the cells cultured for a further 24, 48, or 72 h. Subsequently, 10 μL of CCK-8 reagent was added to each well and the plates incubated for another 2 h, after which absorbance was measured at 492 nm (D value). The inhibitory concentration (IC_50_) and the fractional inhibitory index (FIC) of the CP70 cells were calculated; FIC = IC_50_ drugs in combination/IC_50_ [14]. Reversal of drug resistance was calculated as the inverse of this ratio. Concurrently, CP70 cells were treated with 1, 2, 4, 8, 16, 32, or 64 μg/mL cisplatin and 0.01, 0.1, 1, or 10 mM metformin, and the D_492_ values measured after 24 h. Five wells were tested per concentration, and the experiment was repeated independently 3 times.

### Cell cycle and apoptosis assessment by flow cytometry and annexin V/propidium iodide (PI) double-staining

The A2780/CP70 cells were grown to logarithmic growth phase, and the culture medium replaced with medium containing final concentrations of metformin of 0.01, 0.1, 1, or 10 mM for the cell cycle experiments and 10 mM, 50 mM, or 100 mM metformin for the apoptosis experiments. These concentrations were based on a preliminary experiment that showed when the concentration of metformin increased above 10 mM the degree of apoptosis decreased. Cells were then cultured for 48 h, and single cell suspensions were prepared. PBS was used to wash cells 3 times, after which cells were fixed in 70% methanol overnight at 4 °C. Thereafter, the fixed cells were washed twice with PBS, and 500 μL PI staining solution (500 μL staining buffer containing 25 μL 20 × PI, and 10 μl 50 × RNAse A) added, and cells were incubated for 30 min at 37 °C in the dark. BIOLISA flow cytometry was used to analyze the samples at an excitation wavelength of 488 nm and emission wavelength 546 nm (λmax = 478 nm) [15]; the cell cycle was analyzed using CellQuest software, and the experiment was repeated 3 times.

For annexin V-FITC/PI double-staining, cells were collected and washed by centrifugation 3 times, after which 1 × binding buffer (500 μL) was used to resuspend cells. Then, 5 μL annexin V-FITC and 1 μL PI (100 μg/mL) was added to cells treated with 10, 50, or 100 mM/ metformin; cells were kept at room temperature in the dark for 15 min, and apoptosis analyzed by flow cytometry [15]. The experiment was repeated 3 times.

### Western blotting

Cells exposed to cisplatin in the presence and absence of different concentrations of metformin, as well as to the inhibitor LY294002, were lysed. Protein concentrations of cell lysates were determined using a BCA kit. Equal amounts of total cell-lysate protein were electrophoretically separated by SDS-PAGE, and proteins then transferred to a nitrocellulose membrane. Membranes were blocked in TBST containing 5% skim-milk powder for 2 h, after which they were exposed to the primary antibodies overnight at 4 °C. The antibodies were used at the following dilutions: anti-human MRP2 1:5000, anti-IGF1 1:1000, Anti-phospho-IGF1 1:2000, anti-IGF1R 1:1000, anti-phospho-IGF1R 1:500, anti-AKT 1:3000, anti-phospho-AKT 1:1000, and anti-human GAPDH 1:5000. Membranes were then washed in 1 × TBST 3 times for about 10 min each, after which they were incubated with the secondary antibody at 37 °C for 2 h. Membranes were then again washed in 1 × TBST 3 times for about 10 min each. Proteins were visualized using enhanced chemiluminescence reagents. Bands on X-ray films were scanned using a film scanner, and values normalized to the beta-actin gray value to provide the relative intensity ratio. CP70 cells were treated with the same protocol, as well as the extraction of proteins. Therefore, the beta-actin gray was same in CP70 cells. Western blotting experiments were repeated 3 times.

### Real-time fluorescence quantitative PCR detection of gene expression

Cisplatin sensitive– and resistant cells treated with different concentrations of metformin for 24 h were lysed with RIPA buffer and total RNA extracted for real-time fluorescence quantitative PCR. RNA was reverse transcribed and the target genes *MRP2*, *AKT*, *IGF1*, and *IGFR* amplified. The housekeeping gene *GAPDH* was used as an internal reference. The primer sequences were based on the following NCBI reference sequences *MRP2*: NM_000392.4, *IGF-1*: NM_001111283.2, *IGF1R*: NM_000875.4 and *AKT*: NM_005163.2 and amplification conditions used were as follows. *MRP2*: 5′-CACCATAAAGGACAACATCCTT-3′, and 5′-AGGCTGATCCGCTGCTTCTG-3′ (product size: 174 bp). PCR amplification conditions: 94 °C for 120 s, followed by 45 cycles consisting of 60 °C for 30 s, 56 °C for 20 s, and 68 °C for 30 s.

*IGF1*: 5′-TCAGCAGTCTTCCAACCCAA-3′ and 5′-AAGGCGAGCAAGCACAGG-3′ (product size: 118 bp) and *IGF1R*: 5′-GCTCAACGCAGGGAACTAC-3′ and 5′-CACTATCAACAGAACCGCAAT-3′ (product size: 160 bp). PCR amplification conditions: 95 °C for 10 min, followed by 38 cycles consisting of 95 °C for 10 s, 58 °C for 20 s, and 68 °C for 40 s.

*AKT*: 5′–GGACAACCGCCATCCAGACT-3′ and 5′–GCCAGGGACACCTCCATCTC-3′ (product size: 121 bp). PCR amplification conditions: 95 °C for 5 min, followed by 35 cycles consisting of 94 °C for 30 s, 58 °C for 30 s, and 72 °C for 40 s.

Amplification products were analyzed using a 7500/7500 Fast Real-Time PCR System. The relative expression of target genes against the reference gene was obtained from 2^-△△Ct^ values.

### Nude mice model

Animal care and experiments were approved by the Institute Animal Care and Use Committee of the First Affiliated Hospital of Zhengzhou University. Thirty-six female BALB/C-nu nude mice 4–6 weeks of age were selected and weighed. A suspension (0.3 mL) of cultured cells (containing about 5 × 10^6^ CP70 or A2780 cells) were injected s.c. into the right flank of the nude mice (*n* = 12 for CP70, and *n* = 12 for A2780). As control group (*n* = 6), mice were injected s.c. with saline. Mice were all provided with the same diet and were treated with 3 mg/kg body weight cisplatin (*n* = 6 for CP70 cells, and *n* = 6 for A2780 cells) or with 3 mg/kg body weight cisplatin and 200 μg/mL metformin (*n* = 6 for CP70 cells, and *n* = 6 for A2780 cells), in the drinking water. The metformin dose was used according to a previous study [16]. Tumors were allowed to grow for 3 weeks until they reached about 8 mm in diameter.

Tumor volume changes were monitored every 3 days with a vernier caliper, and the longest diameter (a) and shortest diameter (b) measured. From this, the tumor volume (V) was calculated according to the formula V (mm^3^) = 1/6∏ab^2^, and rate of inhibition of the tumor growth compared, according to this formula: tumor growth inhibition rate = (average tumor volume)/control group average tumor volume × 100%. The growth of the nude mice (body weight) was also assessed and compared.

### Statistical analysis

Independent experiments were conducted 3 times and the mean and standard deviation (SD) were determined. Statistical software (SPSS17.0) was used to compare data with analysis of variance (ANOVA) and least square difference (LSD) test, with *P* < 0.05 denoting a statistically significant difference.

## Results

### Drug-resistance index of ovarian cancer cells

The CCK-8 assay showed that different concentrations of cisplatin affected the growth of ovarian cancer cells after 24 h. Cell growth was inhibited to different extents; the FIC of the cisplatin-resistant cell line CP70 cells was 9.34 times that of the parental A2780 cell line. The IC_50_ value of CP70 cells decreased with the increase in metformin concentration, and the differences between the untreated group and the 1 and 10 mM metformin-treated groups were statistically significant (*P* < 0.05). These results suggested that metformin could improve the sensitivity to cisplatin in cisplatin-resistant ovarian cancer cells in a concentration-dependent manner (Table 1, Additional file 1: Figure S1).

**Table 1:** Resistance of ovarian cancer cells to cisplatin after metformin treatment

Cells	Cisplatin IC_50_ (μg/mL)	Fractional inhibitory index
A2780	3.12 ± 0.048	
CP70		
0 mM/metformin	29.16 ± 0.805	9.34
0.01 mM/ metformin	26.43 ± 0.437	8.45
0.1 mM/metformin	23.52 ± 0.432	7.53
1 mM/metformin	19.26 ± 0.215*	6.17
10 mM/ metformin	10.04 ± 0.327*	3.23

*P < 0.05 compared with 0.01 mM metformin

### CP70 cell cycle and apoptosis

**Fig. 1:**
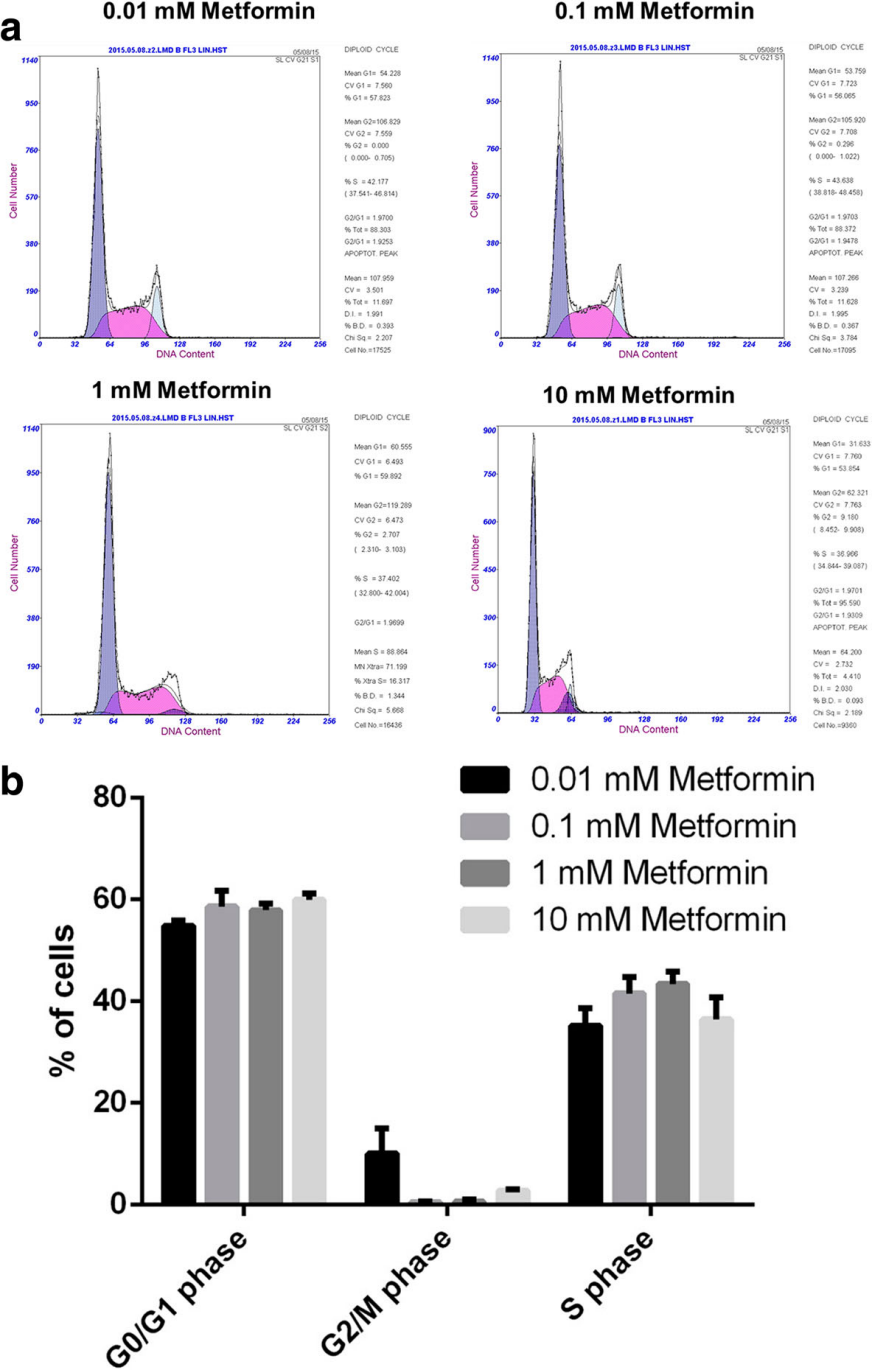
Flow cytometric analysis of CP70 cells treated with different concentrations of metformin for 48 h. **a** different flow cytometry traces according to metformin concentration administered. **b** Analysis and comparison of the results

Flow cytometry revealed that treatment of CP70 cells with 0.01, 0.1, 1, or 10 mM/ of metformin for 48 h influenced the cell cycle distribution, with a dose-dependent effect on the percentage of cells in the G2/M phase. The difference was significant for treatment with 0.1 mM, 1 mM, and 10 mM metformin compared to treatment with 0.01 mM (*P* < 0.05 Fig. 1).

**Fig. 2:**
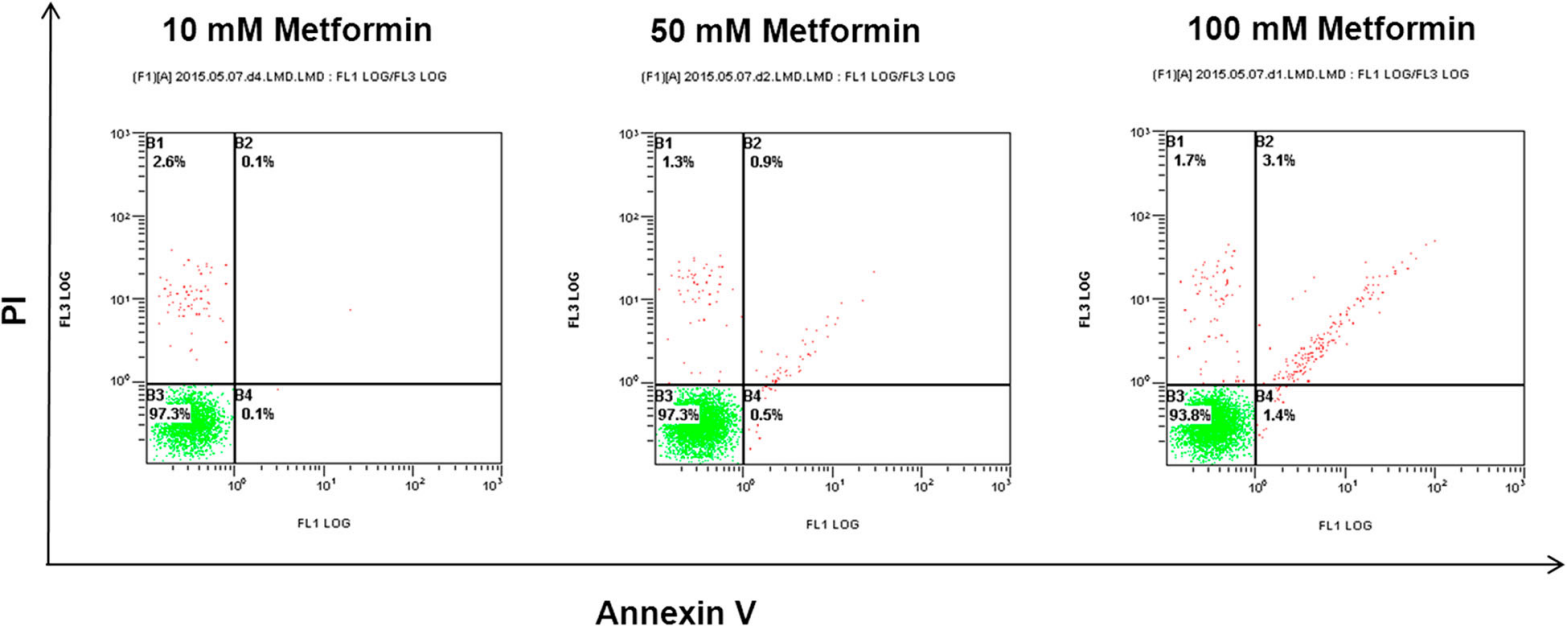
Effects of metformin treatment for 48 h on early apoptosis rates in CP70 cells. The charts are representative examples from one of the experiments. Note: The upper left quadrant: detection error; right upper quadrant: the late apoptotic cells and dead cells; left lower quadrant: normal cells; right lower quadrant: early apoptotic and natural apoptotic cells

The annexin V/PI double-staining method was used to observe apoptosis in CP70 cells treated with metformin for 48 h. CP70 cells treated with 10 mM concentration showed an early apoptosis rate of 0.09 ± 0.12%; treatment with the 50 mM concentration resulted in an early apoptosis rate of 0.46 ± 0.27%, and that with 100 mM, in a rate of 1.25 ± 0.41% as seen in the right lower quadrant of the figures (Fig. 2). There was a statistically significant difference in the early apoptosis rate between cells treated with 100 mM and 10 mM metformin (*P* < 0.05; Fig. 2).

### Effects of metformin on protein expression in CP70 cells

**Fig. 3:**
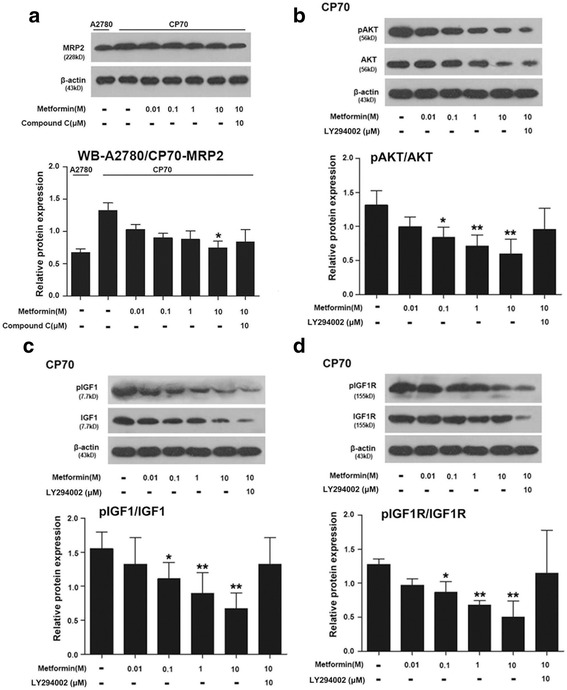
Metformin’s effect on MRP2 (**a**), AKT (**b**), IGF1 (**c**), and IGFR1 (**d**) protein expression in the ovarian cancer cell line CP70. The beta-Actin bands were same in CP70 cells. * shows P < 0.05 between cells untreated with metformin and the treated cells; ** shows *P* < 0.01 between cells untreated with metformin and the treated cells

The expression levels of AKT, IGF1R, and IGF1 in the IGF signaling pathway of cells after 48 h of treatment were assessed using western blotting (Fig. 3). The expression of IGF1, IGF1R, and AKT and their phosphorylated forms were analyzed and expressed as a ratio. This showed that expression of phospho-AKT, phosphor-IGF1R, and phosphor-IGF1 decreased with the increase in the concentration of metformin; the relative expression levels of all three phosphor-proteins were significantly different to those in the untreated cells in the 0.1, 1, and 10 mM metformin groups; all *P* < 0.05. In the 10 mM metformin +10 mM LY294002 group the phosphor-proteins increased compared to the 10 mM metformin cells for all three proteins. Expression levels of MRP2 in cells treated with different concentrations of metformin also decreased from the levels in the untreated group and was significantly different at 10 mM metformin (*P* < 0.05), but did not reach the levels of expression in A2780 cells.

### Gene expression in CP70 cells

**Fig. 4:**
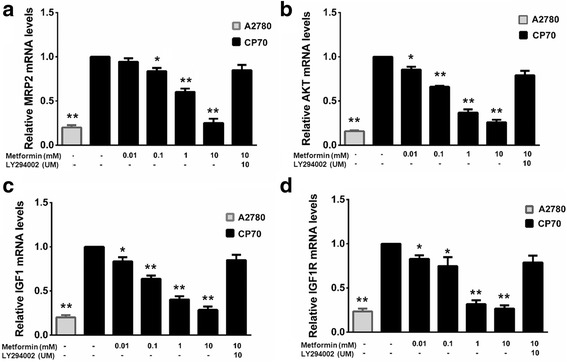
Quantitative real-time PCR of results showing *MRP2* (**a**)*, AKT,* (**b**) *IGF1* (**c**)*,* and *IGFR1* (**d**) expression in CP70 ovarian cancer cells. * shows *P* < 0.05 between untreated CP70 cell and the other cell groups; ** shows *P* < 0.01 between untreated CP70 cells and the other cell groups

Metformin affected *MRP2*, *IGF1*, *IGF1R*, and *AKT* expression in a concentration-dependent manner, thereby inhibiting CP70 cell proliferation activity. Expression levels in cells treated with different concentrations of metformin were statistically significantly different between the untreated group and the 0.1 mM treated groups for each mRNA (Fig. 4). In the 10 mM metformin +10 mM LY294002 groups the expression of all four mRNAs increased compared to the 10 mM metformin cells. The A2780 cell line showed significantly lower expression than the CP70 cells for all the mRNAs studied (*P* < 0.01).

### Inhibition of ovarian carcinoma in nude mice by metformin

**Fig. 5:**
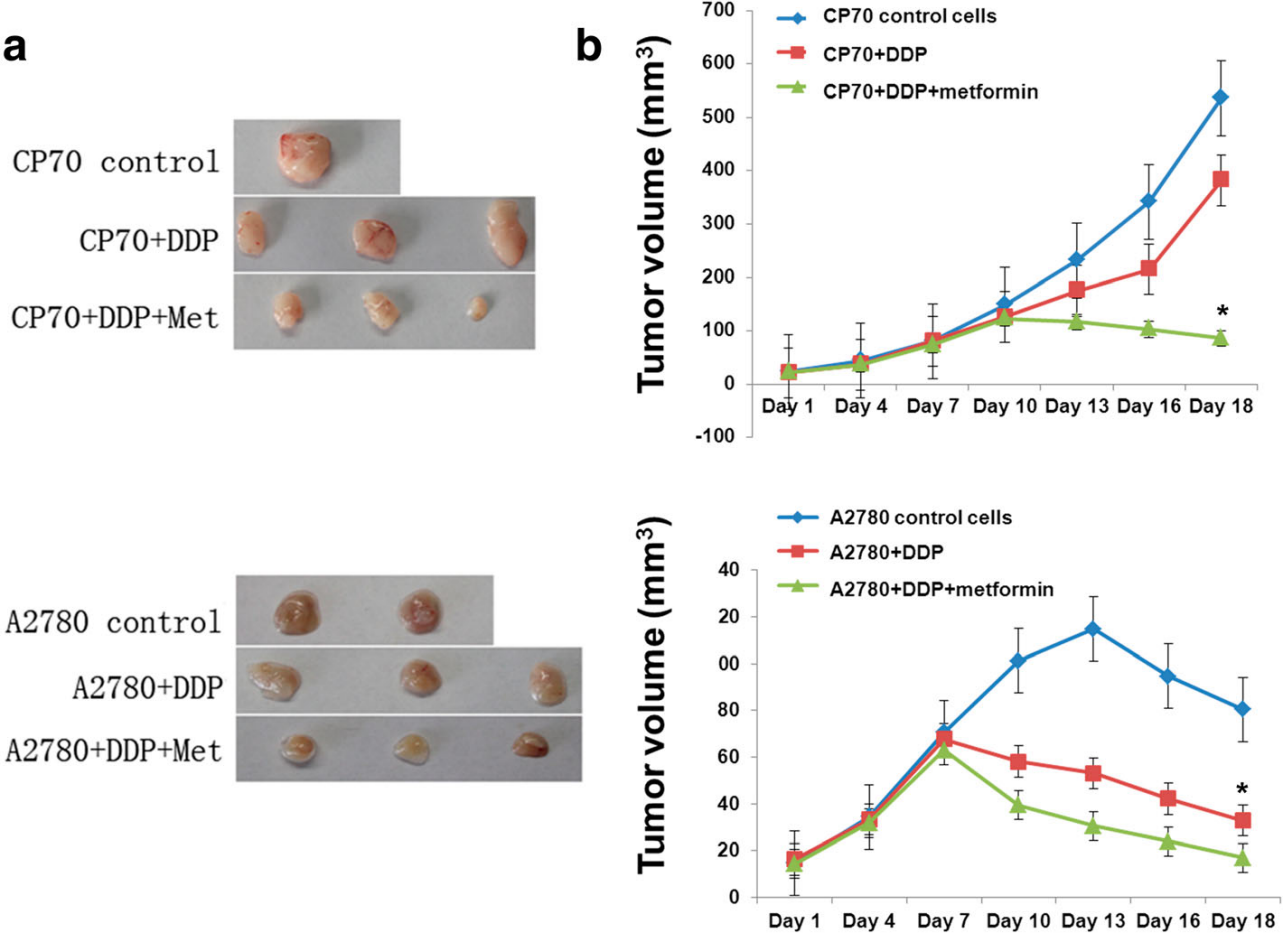
Tumor sizes in CP70 and A2780-inoculated nude mice. **a** Representative images of the excised tumors from the six mice in each group. **b** Tumor volume increases over time with different treatments. * shows *P* <0.05 between CP70 and A2780 control group and metformin treatment group

In both the CP70 and A2780 control groups of nude mice, at 18 days the tumor mass was significantly greater (536.2 ± 12.6 mm^3^ for CP70 and 80.5 ± 1.46 mm^3^ for A2780 cells) than that in the cisplatin (DPP)-treatment groups (382.1 ± 68.7 mm^3^ for CP70 cells and 32.7 ± 9.1 mm^3^ for A2780 cells; Fig. 5). At 18 days the tumor volume in the CP70 + DDP group was greater than that in the CP70 + DDP + metformin group (86.2 ± 51.6 mm^3^). Similarly, the tumor volume in the A2780 + DDP group was greater than that in the A2780 + DDP + metformin group (16.9 ± 5.5 mm^3^).

The growth curves of the tumors in nude mice are shown in Fig. 5. In the CP70 group and CP70 + DDP group, tumor growth showed a rising trend over time, but the tumor growth in CP70 + DDP group was slower than that in the control group. In the CP70 and DDP and metformin group, the tumor size began to decrease after the fourth measurement, and the tumor growth was effectively inhibited.

In the A2780 control group, tumor growth first increased and then decreased over the first 5 time-points. In both the cisplatin-treated groups, the tumor size increased over the first 3 time-points, and then began to decline, with the decline in the A2780 + DDP group + metformin group being greater than that in the A2780 + DDP group.

The body weight of nude mice in each group was determined every 3 days. The body weight in 3 groups of nude mice increased over time. In the CP70 + DDP + metformin group the increase in weight started to plateau at day 9 in group CP70 + DDP it started to plateau at day 13, but CP70 control mice continued to increase at day 17. However, the CP70 + DDP + metformin group had a higher body weight at the beginning of the study despite their randomization into groups (Additional file 1: Figure S2A). In the A2780 group of nude mice, body weight increased over time, with body weight in the control group > CP70 + DDP + metformin group > CP70 + DDP group (Additional file 1: Figure S2B).

### Tumor inhibition rate

The tumor growth inhibition rate was calculated by comparing the tumor volume in the control group and in the treatment groups. In the CP70 mice, the CP70 + DDP group showed a tumor-inhibition rate of 28.73%. The combined use of metformin and cisplatin resulted in a tumor-inhibition rate of 83.93%, which was 55.2% higher than achieved using cisplatin alone. The inhibition rate in the A2780 + DDP was 59.16%, while that in the A2780 + DDP + metformin group was 79.03%; thus, the inhibition rate of the combined therapy was 19.87% higher than that cisplatin alone. These results demonstrate that co-treatment with cisplatin and metformin can inhibit the growth of epithelial ovarian cancer significantly more than can cisplatin alone (*P* < 0.05; Table 2, Additional file 1: Figure S3).

**Table 2:** Tumor inhibition rate in the CP70 group and A2780 group

Group		Tumor-inhibition rate	*P*-value
CP70	CP70 + DDP group	28.73%	< 0.05^a^
	CP70 + DDP + Metformin group	83.93%	
A2780	A2780 + DDP group	59.16%	< 0.05^b^
	A2780 + DDP + Metformin group	79.03%	

^a^ *P* < 0.05, compared with 0.01 mM CP70 group

^b^ *P* < 0.05, compared with 0.01 mM A2780 group

## Discussion

Currently, the main treatment for ovarian cancer is surgery and chemotherapy. The prognosis of ovarian cancer is largely determined by the effect of chemotherapy, and chemotherapy resistance is the main reason for treatment failure. Drug resistance of the tumor is due to multiple factors, such as decreased intracellular drug concentration, an impaired cellular detoxification system, a change in the drug target, an abnormal DNA damage repair system, genetic modifications, and abnormalities in the regulation of apoptosis.

Multidrug resistance (MDR) refers to the phenomenon of cross-resistance of tumor cells to the structure and mechanism of anti-tumor drugs, after the tumor cells have gained resistance to a type of anti-tumor drug [17]. One protein that has been implicated in cisplatin resistance is MRP2, which transports cytotoxic agents from cells and is expressed at higher levels when cell lines become cisplatin resistant [10–12]. Other efflux systems have also been implicated in MDR. Studies have shown that P-gP protein, a transmembrane P-glycoprotein encoded by *MDR1,* contributes to drug-resistance in cancer cells [18]. P-gP is one of the earliest proteins known to be related to MDR [19] and was shown to contain an ATP-binding domain, and have an energy-dependent drug efflux function [20]. Interestingly, in a mirror of this study inhibition of the c-Jun N-terminal kinase (JNK) signaling pathway enhanced the sensitivity of hepatocellular carcinoma cells to cisplatin by down-regulating the expression of P-gp [21]. Similarly, a previous study has shown that metformin interacts with the IGF pathway, and induces apoptosis and inhibition of proliferation and migration of uterine serous carcinoma cell lines [22], so this led us to investigate the mechanism of metformin increasing resistance in ovarian cells with attention on MRP2.

Many epidemiological studies have suggested that metformin, a first-line drug for the treatment of type 2 diabetes mellitus, may reduce the incidence of gastric cancer, liver cancer, colon cancer, breast cancer, prostate cancer, and other cancers in patients with type 2 diabetes [23–25]. Moreover, a recent retrospective study found that the use of metformin in diabetic patients can reduce the risk of pancreatic cancer by about 46% [26]. Based on these clinical studies, an increasing number of basic research studies has reported that metformin has a strong direct inhibitory effect on the growth of many malignant tumors in vitro and in vivo [27–29]. Kiyohito et al. found that metformin has a significant inhibitory effect on the proliferation of human gastric cancer cell lines such as MKN1, MKN45, and MKN74, and there was a positive correlation between the inhibition rate and metformin dose [30] in in vitro experiments on lung adenocarcinoma, colon cancer, prostate cancer, and other malignant tumor cell lines [31, 32]. In an animal experiment, Algire et al. demonstrated that systemic administration of metformin can reduce the incidence of a high fat diet-induced pancreatic cancer in hamsters [33]. Metformin also has an influence on an ovarian cancer cells. Our results showed that 0.01, 0.1, 1, and 10 mM metformin could improve the sensitivity of ovarian cancer cells to cisplatin; MDR reversal was concentration dependent. However, as in most of the other cell based studies these levels of metformin are much higher than the therapeutically relevant plasma concentration of metformin (20 μmol/L) [13]. Therefore, further research is needed to evaluate whether metformin would be directly relevant clinically.

The abnormal regulation of cell cycle is an important mechanism leading to abnormal cell proliferation. Transition from the G0/G1 phase to S phase is a key node in the cell cycle; if cells are blocked at the G0/G1 stage, cell proliferation is slowed [34]. Cyclin D1 regulates the G0/G1 to S phase transition, elevates the expression of cyclin D1 to significantly shorten the G1 phase, and accelerates G1 to S phase transition, thus speeding up cell cycle progression [35]. In a study of the effect of metformin on prostate cancer cells, Ben et al. showed that metformin can reduce intracellular cyclin D1 expression and cell cycle arrest in the G0/G1 stage, thereby inhibiting cell proliferation [27]. Chen et al. found that metformin inhibits mTOR by activating AMPK, which leads to phosphorylation of downstream target molecules, and eventually blocking liver cancer cells in G0/G1 phase [36]. In our study, treatment of CP70 cells with 0.01, 0.1, 1, and 10 mM metformin for 48 h changed the cell cycle distribution, and the percentage of cells in the G2/M phase was significantly different between the 0.01 mM and 10 mM groups, with many fewer cells in G2/M when treated with metformin. Although the differences were not significant for the numbers of cells in G0/G1 or S phase, we think that these subtle increases in numbers with metformin treatment may have an influence on the number of cells in G2.

Tumor development is not related only to abnormal proliferation of cells, but is also closely related to abnormal cell apoptosis, an active, programmed cell death process that occurs under certain physiological or pathological conditions. Cell apoptosis is regulated by many signaling molecules, which are closely related to tumor occurrence, development, treatment, and prognosis. Buzzai et al. found that metformin can promote early apoptosis in colon cancer cells, and inhibit the growth of such cells [37]. Wu et al. demonstrated that metformin can promote early apoptosis of lung cancer cells [31]. In this study, we used annexin V/PI double-staining to detect the effect of metformin in CP70 cells after 48 h, to determine the rate of change of early apoptosis. We found that with an increase in the concentration of metformin, the CP70 cell apoptosis rate increased gradually.

Insulin-like growth factor binds to 2 receptors, IGF1R and IGF2R, but IGF2R is a tumor suppressor [38]. Insulin-like growth factor has been shown to inhibit apoptosis induced by chemotherapy, and to activate the PBK-AKT signaling pathway. The inhibitory effect of the insulin-like growth factor receptor has been shown to increase the efficacy of carboplatin etoposide. It has been suggested that an increase in IGF1 levels is associated with malignant and non-malignant tumors, and can promote the proliferation and division of tumor cells. It is possible to reverse the effect of IGF1 by reducing the levels of insulin and insulin-binding protein, which may be the mechanism underlying its anti-tumor proliferative effects [39]. The PI3K/AKT and Ras/Raf/MEK/ERK signaling pathways are 2 important pathways by which mTOR signals. Variation in PI3K is seen in various tumors with abnormal activation of the tumor suppressor PTEN [39], for example, in pancreatic cancer, colon cancer, bladder cancer, cervical cancer, uterine endometrial cancer, ovarian cancer, and liver cancer that harbor Ras mutations [40].

In this study, we used western blotting and PCR and found that metformin treatment of CP70 for 48 h significantly decreased the expression of phosphorylated forms of IGF1, IGF1R, and AKT in a concentration-dependent manner, compared to the control group. It can therefore be concluded that the inhibition of growth of CP70 ovarian cancer cells in vivo by metformin occurs via the inhibition of MRP2 expression, which was clearly seen at mRNA level with significant decreases at 0.1, 1 and 10 mM metformin, but less obvious at protein level because it was only significant at 10 mM metformin. These differences may be due to the time taken to degrade the MRP2 protein, but there is a definite role for protein synthesis in regulation [41]. A reduction in cancer cell division and proliferation due to decreased endogenous fatty acid synthesis results in aberrant metabolism in cancer cells, and will prevent the cell entering G2/M phase, thus inhibiting proliferation of CP70 cells. Concurrently, metformin also induces apoptosis in CP70 cells, thus further inhibiting their proliferation.

In humans, metformin can cause gastrointestinal problems and a resulting weight loss [42]. The weight of the mice in this study increased over the study period, the mice treated with metformin with CP70 induced tumors started to plateau earlier than the other groups, but as the weight was higher in this group originally this may be a natural growth pattern. The mice in the A2780 induced tumor group did not show any obvious differences in weight gain during the study.

Furthermore, we showed in experiments in nude mice and in ovarian cancer cell lines, that MRP2 expression was higher in cisplatin-resistant CP70 cells than in the cisplatin-sensitive A2780 cells. Moreover, we showed that metformin could inhibit the growth of ovarian cancer in mice, significantly more than cisplatin alone.

This study has some limitations, our signaling studies used metformin (1 or 10 mmol/L) at a level that exceeds its therapeutic plasma concentration (Cmax20μmol/L) [13]. The mouse model received a dose of metformin according to the doses administered in a previous study [15], but we did not measure the levels in serum to establish the similarities with the concentrations used in vitro. Cisplatin is generally administered intravenously at 60-80 mg/m^2^, while 3 mg/kg was given in the animal experiments, therefore, the dosage is lower the clinical drug dosage threshold. Further experiments are therefore needed to investigate the effect of a therapeutically relevant plasma concentration of metformin (20 μmol/L) on AKT phosphorylation and chemoresistance in ovarian cancer. The investigations should be expanded into other cell lines to ensure that these results are the same in other ovarian cancer models. We also did not have positive or negative controls in the mouse histology experiments so these results may be misleading.

## Conclusions

In summary, the mechanisms underlying the anti-tumor effects of metformin are complex, but involve at least inhibition of MRP2 expression and promotion of apoptosis. Whether other molecular mechanisms are involved should be investigated in future.

## Additional file

Additional file 1: Figure S1. Cell growth inhibition curves of A2780 cells and CP70 cells undergoing different treatments involving cisplatin (DDP) and metformin. **Figure S2.** Weight of the mice inoculated with CP70 and A2780 that underwent different treatments involving cisplatin (DDP) and metformin cells over the study period. **Figure S3.** Tumor mass at 18 days of the mice inoculated with CP70 and A2780 cells and undergoing different treatments involving cisplatin (DDP) and metformin. (PDF 251 kb)

## Abbreviations

DDP: Cisplatin
ERCC1: Excision repair cross-complementation 1
FBS: Fetal bovine serum
LSD: Least square difference
MRP2: Multidrug resistance associated protein 2
PI: Propidium iodide
SD: Standard deviation
