# Supplementary material for: Inhibition of the IGF signaling pathway reverses cisplatin resistance in ovarian cancer cells
Source: BMC Cancer. 2017 Dec 14;17:851. doi: 10.1186/s12885-017-3840-1 (PMC5731066; doi:10.1186/s12885-017-3840-1)

Supplementary figures

Figure S1. Cell growth inhibition curves of A2780 cells and CP70 cells undergoing different treatments involving cisplatin (DDP) and metformin.

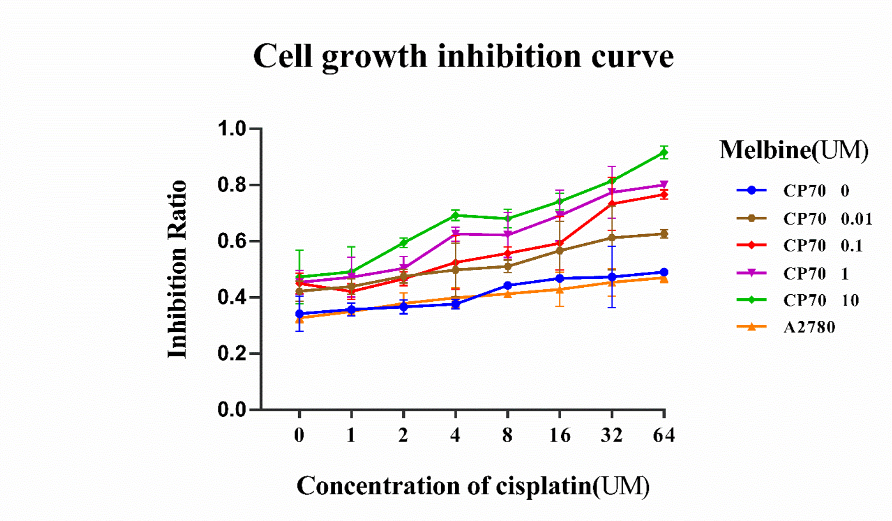

Figure S2. Weight of the mice inoculated with CP70 and A2780 that underwent different treatments involving cisplatin (DDP) and metformin cells over the study period.

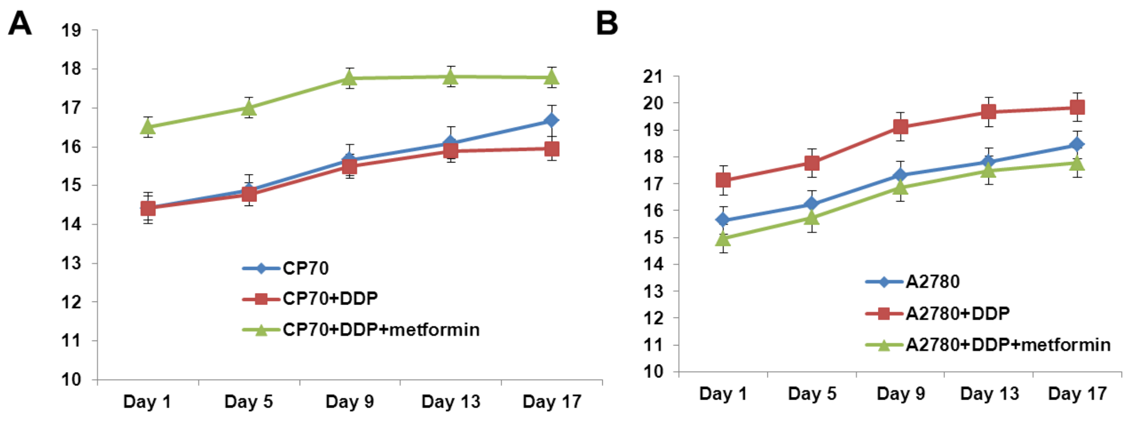

Figure S3. Tumor mass at 18 days of the mice inoculated with CP70 and A2780 cells and undergoing different treatments involving cisplatin (DDP) and metformin.

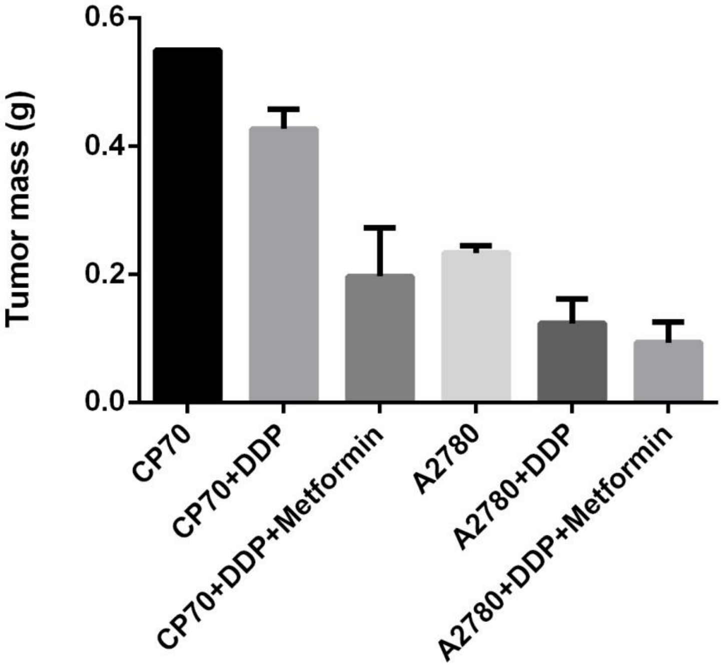

Supplement: Additional file 1: Figure S1. — Cell growth inhibition curves of A2780 cells and CP70 cells undergoing different treatments involving cisplatin (DDP) and metformin. Figure S2. Weight of the mice inoculated with CP70 and A2780 that underwent different treatments involving cisplatin (DDP) and metformin cells over the study period. Figure S3. Tumor mass at 18 days of the mice inoculated with CP70 and A2780 cells and undergoing different treatments involving cisplatin (DDP) and metformin. (PDF 251 kb) [file 12885_2017_3840_MOESM1_ESM.pdf]
